# Analyses of functions of an anti-PD-L1/TGFβR2 bispecific fusion protein (M7824)

**DOI:** 10.18632/oncotarget.20680

**Published:** 2017-09-08

**Authors:** Caroline Jochems, Sarah R. Tritsch, Samuel Troy Pellom, Zhen Su, Patrick Soon-Shiong, Hing C. Wong, James L. Gulley, Jeffrey Schlom

**Affiliations:** ^1^ Laboratory of Tumor Immunology and Biology, Center for Cancer Research, National Cancer Institute, National Institutes of Health, Bethesda, MD, USA; ^2^ EMD Serono, Rockland, MA, USA; ^3^ NantWorks, Culver City, CA, USA; ^4^ Altor BioScience Corporation, Miramar, FL, USA; ^5^ Genitourinary Malignancies Branch, Center for Cancer Research, National Cancer Institute, National Institutes of Health, Bethesda, MD, USA

**Keywords:** anti-PD-L1/TGFβR2, M7824, checkpoint inhibitors, immunotherapy, anti-PD-L1

## Abstract

M7824 (MSB0011359C) is a novel first-in-class bifunctional fusion protein consisting of a fully human IgG1 anti-PD-L1 monoclonal antibody (with structural similarities to avelumab) linked to the extracellular domain of two TGFβ receptor 2 (TGFβR2) molecules serving as a TGFβ Trap. Avelumab has demonstrated clinical activity in a range of human cancers and has been approved by the Food and Drug Administration for the therapy of Merkel cell and bladder carcinomas. Preclinical studies have shown this anti-PD-L1 is capable of mediating antibody-dependent cell-mediated cytotoxicity (ADCC). In the studies reported here, it is shown that M7824 is also capable of mediating ADCC of a wide range of human carcinoma cells *in vitro*, employing natural killer (NK) cells as effectors, albeit not as potent as anti-PD-L1 employing some tumor cells as targets. The addition of the IL-15 superagonist fusion protein complex ALT-803 enhanced the ADCC capacity of both anti-PD-L1 and M7824, and to levels that both agents now demonstrated similar levels of ADCC of tumor cells. TGFβ is a known immunosuppressive entity. Studies reported here show TGFβ1 induced reduction of several NK activation markers as well as reduction of endogenous NK lytic activity and NK-mediated ADCC of tumor cells. These phenomena could be reduced or mitigated, however, by M7824, but not by anti-PD-L1. M7824, but not anti-PD-L1, was also shown to reduce the immunosuppressive activity of regulatory T cells on human CD4^+^ T-cell proliferation. These studies thus demonstrate the dual functionalities of M7824 and provide the rationale for its further clinical development.

## INTRODUCTION

The use of checkpoint inhibitor monoclonal antibodies (MAbs) has transformed the use of immunotherapy as a major modality in the treatment of several forms of cancer [1–5]. The major class of these checkpoint inhibitors has been either anti-PD-1 or anti-PD-L1 agents, which interfere with the binding of tumor-expressed PD-L1 with PD-1 on T cells, resulting in “releasing the brakes” of otherwise anergized T cells, and consequently enhancing anti-tumor effects. Most of the anti-PD-1/PD-L1 MAbs are either of the IgG4 isotype, or of the IgG1 isotype and engineered to inhibit any potential antibody-dependent cell-mediated cytotoxicity (ADCC) in an effort to eliminate any potential toxicities due to the PD-1 or PD-L1 expression on normal cells. One exception to this is the human IgG1 anti-PD-L1 avelumab (Bavencio®). Avelumab was specifically designed to mediate ADCC in an effort to enable this agent with a second potential anti-tumor mechanism. Recent *in vitro* studies [6, 7] have demonstrated that avelumab indeed has the ability to mediate lysis of a range of human tumor cells using human natural killer (NK) cells as effectors. Clinical studies employing avelumab have now demonstrated clinical benefit in the use of avelumab in a range of human tumors [8–11]. Avelumab has recently been approved by the Food and Drug Administration for the therapy of Merkel cell carcinoma and bladder cancer. Adverse events, above those seen with other anti-PD-1/PD-L1 MAbs, have not been observed [8–11]. Moreover, an extensive interrogation of 123 immune cell subsets in the periphery of patients receiving up to nine cycles of avelumab has shown [12] no statistically significant changes in any immune subset compared to baseline.

With the success achieved with anti-PD-1/PD-L1 MAbs in the treatment of some melanoma patients and approximately 10-20% of patients with some other cancers, the majority of cancer patients with solid tumors are still not experiencing clinical benefit with these agents [5]. One potential reason for this is the existence of immunosuppressive entities in the tumor microenvironment. Prior studies have shown [13–16] that TGFβ, secreted by tumor cells in an autocrine loop, or in a paracrine fashion by immunosuppressive cells or stroma in the tumor microenvironment, can inhibit the anti-tumor activity of effector cells such as NK or T cells.

The studies reported here describe several functions of a novel bifunctional fusion protein consisting of an anti-PD-L1 MAb with structural similarities to avelumab linked to two TGFβ receptor 2 (TGFβR2) molecules, and designated M7824 (MSB0011359C). Preclinical murine studies have shown the anti-tumor activity of M7824 (Lan, manuscript submitted), and a recent dose escalation first-in-human Phase I study [17, 18] has demonstrated evidence of anti-tumor activity with adverse events generally consistent with those of other anti-PD-1/PD-L1 agents.

The *in vitro* studies reported here demonstrate that M7824 maintains its ability to mediate ADCC for a range of human tumor cell types employing NK effectors from both healthy donors and cancer patients, albeit to a lower level than that observed with anti-PD-L1 (avelumab). The exposure of NK cells to the IL-15 superagonist/IL-15Rα-Fc (ALT-803) [19–21] enhanced the ADCC-mediating capacity of both anti-PD-L1 and M7824, but also raised the level of ADCC activity of M7824 to that of anti-PD-L1. Exposure of NK cells to TGFβ was shown to reduce the level of NK activation markers and reduce both NK tumor cell lysis and NK-mediated ADCC. These phenomena were shown to be reversed by M7824 and not by anti-PD-L1. Moreover, the M7824 molecule, and not anti-PD-L1, was shown to reduce the immunosuppressive effect of regulatory T cells (Tregs) on CD4^+^ proliferative activity. In sum, these studies demonstrate the multifunctionality of this novel immunotherapeutic agent.

## RESULTS

### M7824 can induce ADCC

**Figure 1:**
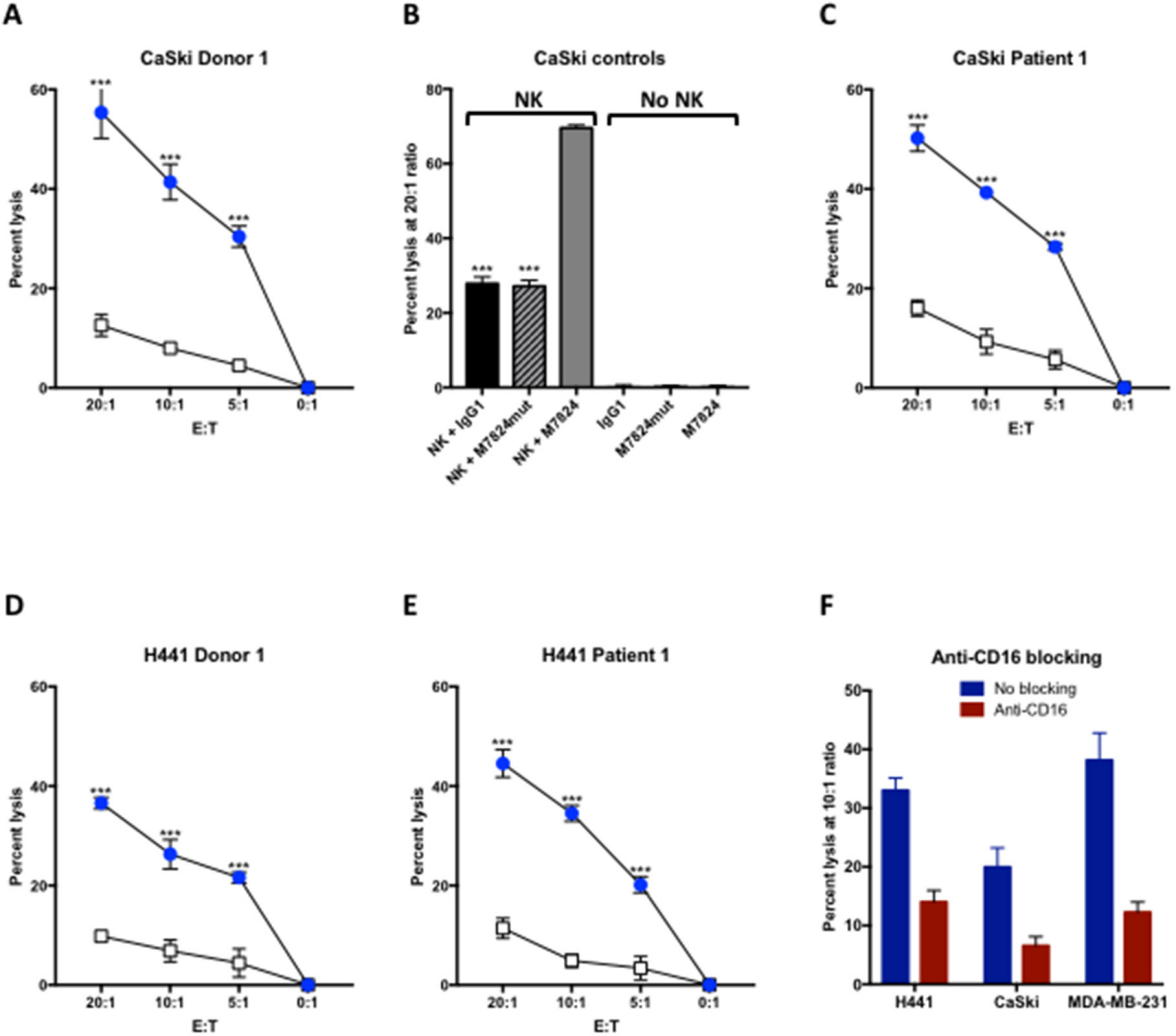
M7824 can mediate ADCC NK cells were isolated from PBMCs from three cancer patients and three healthy donors, rested overnight, and then used in an ^111^In-release 20-hour assay to evaluate NK lysis of tumor cells (with no MAb and with an IgG1 isotype control MAb 1 μg/ml), and ADCC mediated by M7824 (1 μg/ml), as described in Materials and Methods. Panels **A** and **C** show NK lysis (white squares) and M7824-mediated ADCC (blue circles) of the human cervical carcinoma cell line CaSki, using NK cells from one healthy donor (Panel A) and one patient with metastatic breast carcinoma (Panel C). Panel **B** (CaSki controls) shows that the M7824mut, a molecule encompassing a mutant anti-PD-L1 that does not bind to PD-L1 but contains the intact TGFßR2 segment, did not induce ADCC. In addition, neither M7824 nor M7824mut induced lysis of target cells in the absence of NK cells (Panel B, right side). Panels **D** and **E** show NK lysis (open squares) and M7824-mediated ADCC (blue circles) of the human lung carcinoma cell line H441 using NK cells from a healthy donor and a breast carcinoma patient, respectively. Results are shown at several E:T ratios, with mean and standard deviations of triplicate wells. All experiments have been repeated multiple times with similar results. Multiple t-tests were used to compare ADCC with NK cell lysis at each E:T ratio, *** P < 0.001. Panel **F** NK cells isolated from a healthy donor were untreated (blue bars) or pre-incubated for 2 hours with anti-CD16 blocking antibody (50 μg/ml, red bars) before being used as effector cells in NK lysis or M7824-mediated ADCC assays.^111^In-labeled H441 (lung cancer), CaSki (cervical cancer), and MDA-MB-231 (breast cancer) tumor cells were used as target cells with M7824 (1 μg/ml) to mediate ADCC. Results shown are from one healthy donor at an E:T ratio of 10:1, assayed in triplicate wells. The experiment has been repeated with other donors with similar results.

Indium-release assays were performed to determine if M7824 could induce ADCC with NK cells isolated from three healthy donors and three cancer patients as effectors. Representative results are shown in Figure 1, using as targets human cervical carcinoma cells (CaSki, Figure 1A-1C), and human lung carcinoma cells (H441, Figure 1D and 1E), at several different effector to target cell (E:T) ratios. NK lysis (white squares, employing control IgG1 antibody) and ADCC induced by M7824 (blue circles) are shown using NK cells derived from a healthy donor (Figure 1A, 1B and 1D) and a cancer patient (Figure 1C and 1E). For all experiments, control IgG1 and no MAb were used as controls to evaluate NK lysis, and results were similar for all samples analyzed. In contrast to the ADCC induced by M7824, M7824mut, a molecule encompassing a mutant anti-PD-L1 that does not bind to PD-L1, did not enhance tumor cell lysis (Figure 1B, hatched bar). In the absence of NK cells, none of the agents induced lysis of tumor cells (Figure 1B). To demonstrate that the enhanced lysis by NK cells observed with the addition of M7824 involves the ADCC mechanism, anti-CD16 MAb was shown to reduce the lysis of three different human tumor cell lines (Figure 1F). In additional experiments, similar results were observed using NK cells isolated from two additional cancer patients and eight healthy donors; in those experiments M7824-mediated ADCC was seen using as targets six of seven different human tumor cell lines, including CaSki, the breast carcinoma line MDA-MB-231, and lung carcinoma cell lines H441 and HCC4006; lower levels of lysis were seen with the lung carcinoma line H460 or the prostate carcinoma line PC3 as a target (Supplementary Table 1). No ADCC was observed using MCF7 cells as targets, which could be expected since MCF7 does not express PD-L1 (Supplementary Table 1).

### Pretreating NK cells with the IL-15 immunocytokine ALT-803 increased NK lysis and ADCC induced by M7824

**Figure 2:**
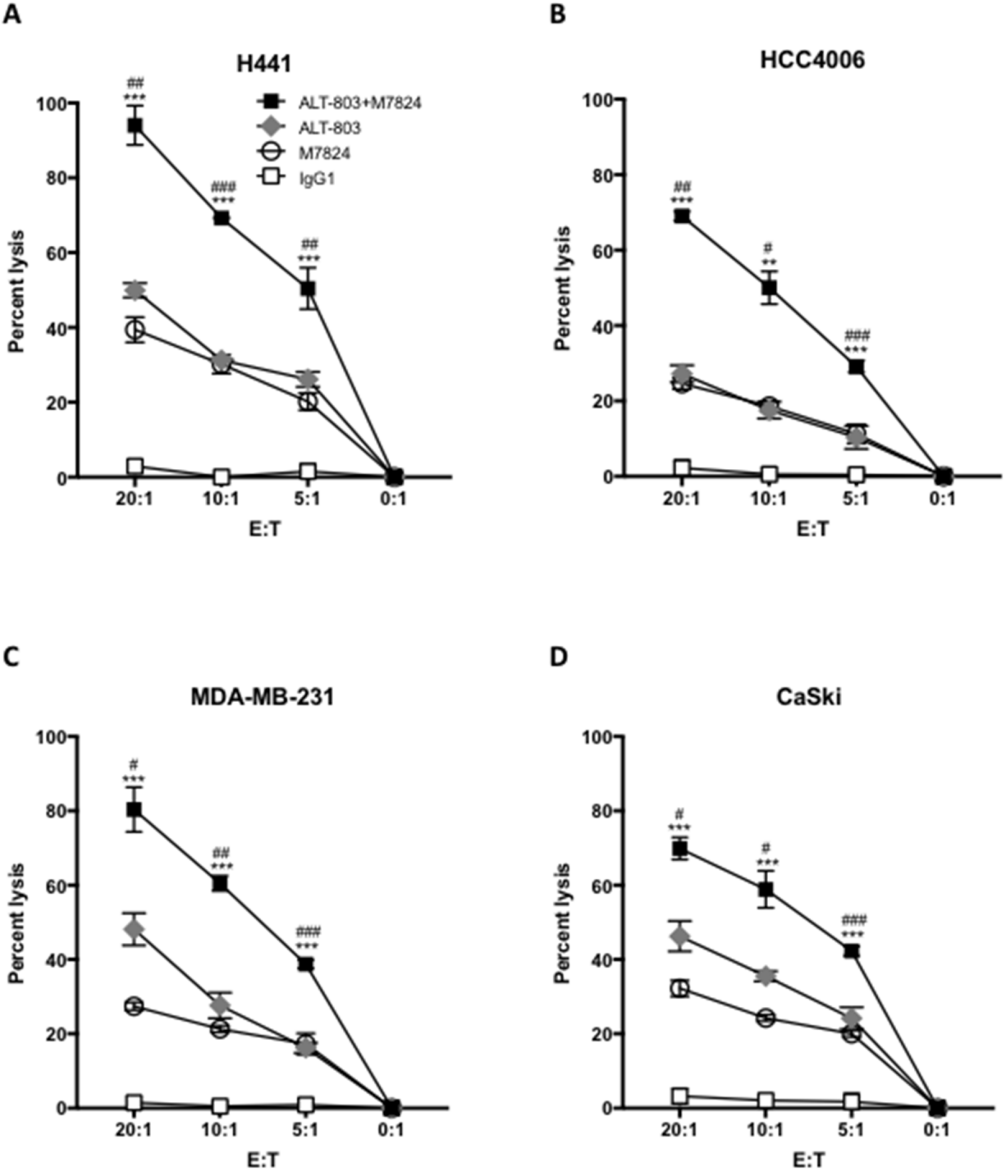
Pretreating NK cells with ALT-803 increases tumor cell lysis and ADCC mediated by M7824 NK cells were isolated from PBMCs from three cancer patients and three healthy donors; NK cells were untreated or treated (24 hours) with ALT-803 (IL-15 superagonist/IL-15RαSushi-Fc fusion complex, 25 ng/ml), and then used in ^111^In-release 20-hour assays to evaluate NK tumor cell lysis (no MAb and control IgG1, 1 μg/ml), and ADCC mediated by M7824 (1 μg/ml), as described in Materials and Methods. Results from one representative cancer patient are shown using as targets four human tumor cancer cell lines: H441 (lung carcinoma, Panel **A**), HCC4006 (lung carcinoma, Panel **B**), MDA-MB-231 (breast carcinoma, Panel **C**), and CaSki (cervical carcinoma, Panel **D**). NK tumor cell lysis is shown as white squares (same results with no MAb and IgG1 control antibody, which is shown); grey diamonds denote tumor cell lysis for ALT-803–treated NK cells. ADCC mediated by NK cells plus M7824 is shown as white circles; black squares denote M7824-mediated ADCC using NK cells treated with ALT-803. Results are shown at different E:T ratios, with mean and standard deviations of triplicate wells, using NK cells from a cancer patient. NK cells from two other cancer patients and three healthy donors showed similar results. Multiple t-tests were used to compare ADCC mediated by M7824 using ALT-803–treated NK cells (black squares) vs. ADCC mediated by M7824 using untreated NK cells (white circles) at each E:T ratio, *** P < 0.001, ** P < 0.01, * *P* < 0.05. In addition, for ALT-803–treated NK cells, we also compared NK lysis (grey diamonds) vs. M7824-mediated ADCC (black squares), ^###^ *P* < 0.001, ^##^ *P* < 0.01, ^#^ *P* < 0.05.

The IL-15 superagonist/IL-15Rα-Fc fusion complex (designated ALT-803) has previously been shown to enhance NK lysis of tumor cells and to augment NK-cell ADCC of B cell lymphomas directed by rituximab [21, 22]. That is also shown in Figure 2 (diamonds). The level of NK tumor lysis observed with the addition of ALT-803 was similar to that observed with the addition of M7824 (Figure 2, circles). The treatment of NK cells with both M7824 plus ALT-803, however, statistically enhanced the level of lysis of all four tumor cell lines analyzed (Figure 2, squares). The use of NK cells isolated from two additional cancer patients and three healthy donors also showed similar results.

### Comparison of ADCC induced by M7824 and anti-PD-L1 MAb with and without pretreatment of the NK cells with ALT-803

**Figure 3:**
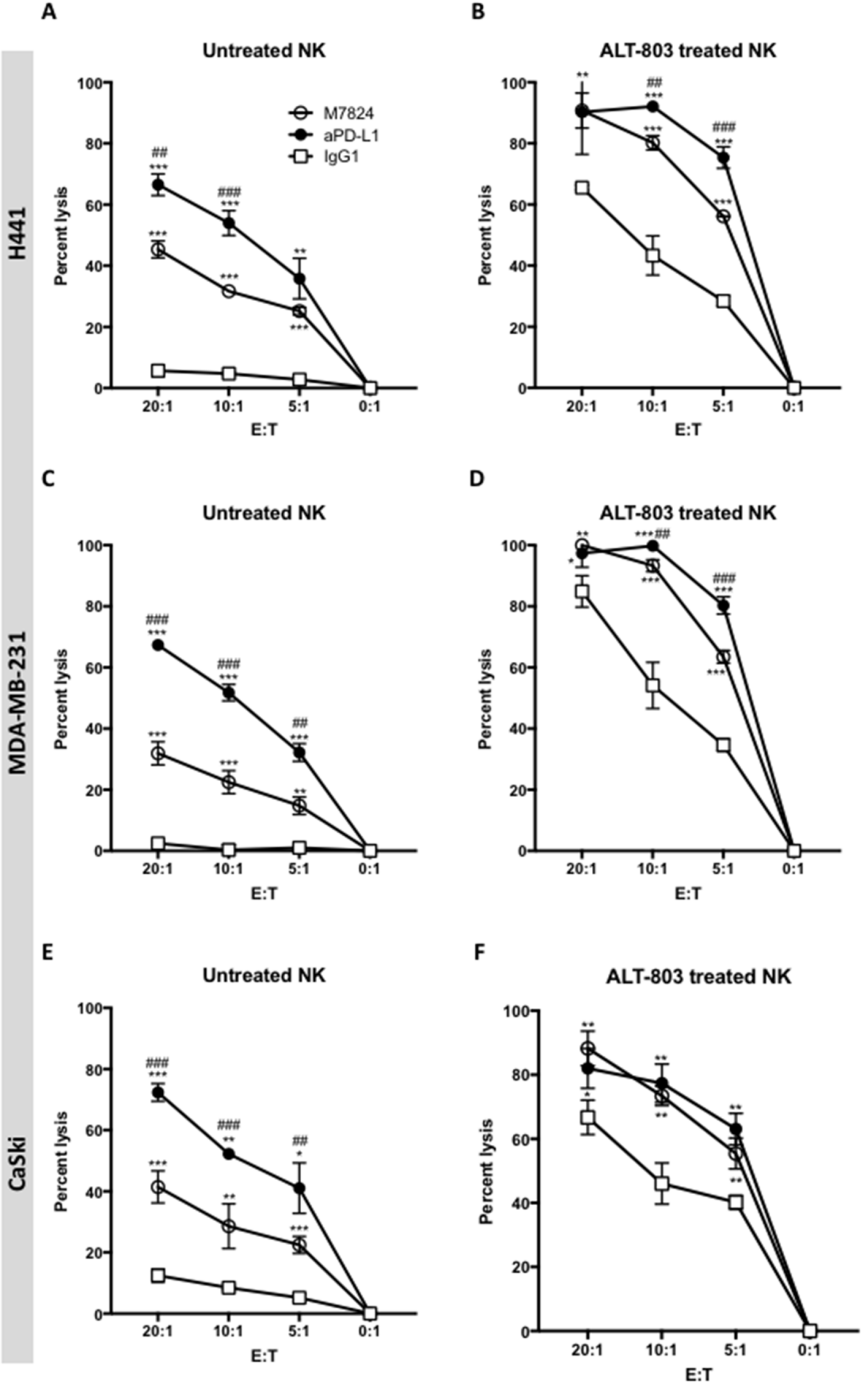
ADCC of tumor cells mediated by M7824 vs. anti-PD-L1, with and without pretreatment of effector NK cells with ALT-803 NK cells were isolated from PBMCs from three healthy donors, rested untreated (Panels **A, C** and **E**) or treated (24 hours) with ALT-803 (Panels **B, D** and **F**), and then used in ^111^In-release 20-hour assays to evaluate NK tumor cell lysis (white squares, IgG1 1 μg/ml) and ADCC mediated by M7824 and anti-PD-L1. ADCC mediated by anti-PD-L1 is shown in black circles and ADCC mediated by M7824 is shown in white circles. Only isotype control IgG1 antibody is shown, since the no MAb control overlapped. Results from one of three healthy donors are shown at different E:T ratios, with mean and standard deviations of triplicate wells using as targets H441 (lung cancer, Panels A and B), MDA-MB-231 (breast cancer, Panels C and D), and CaSki (cervical cancer, Panels E and F). NK cells from the other two donors showed similar results. Results with the use of ALT-803–treated NK cells are shown in Panels B, D, and F. Multiple t-tests were used to compare ADCC mediated by anti-PD-L1 (black circles) vs. NK lysis (white squares), and ADCC mediated by M7824 (white circles) vs. NK lysis (white squares), at each E:T ratio, *** P < 0.001, ** P < 0.01, * *P* < 0.05. In addition, we also compared ADCC mediated by M7824 (white circles) vs. anti-PD-L1 (black circles), ^###^ *P* < 0.001, ^##^ *P* < 0.01, ^#^ *P* < 0.05. Only the significant comparisons are shown.

One component of the M7824 molecule is derived from the anti-PD-L1 molecule avelumab, which has previously been shown to mediate ADCC. Studies were undertaken to compare the ADCC activities of anti-PD-L1 with that of M7824 using NK cells as effectors. As seen in Figure 3 (Panels A, C, and E), anti-PD-L1 demonstrated greater ADCC activity compared to M7824 at all E:T ratios, and for each of the three tumor cell lines used as targets. The addition of ALT-803 enhanced the tumor cell lysis mediated by both anti-PD-L1 and M7824. With the addition of ALT-803, however, the level of tumor cell lysis employing M7824 was now similar to that observed by anti-PD-L1 at each E:T ratio (Figure 3B, 3D, and 3F). It should be noted that these studies were conducted at equimolar concentrations of M7824 and anti-PD-L1 (1 μg/ml and 0.8 μg/ml, respectively). Figure 3 shows representative results using NK cells as effectors from a healthy donor; similar results were observed using NK cells from two additional donors and using two additional tumor cell lines as targets. Supplementary Figure 1 shows the use of H460 and HCC4006 lung carcinoma cell lines as targets with similar results.

### M7824 induces ADCC lysis of tumor cells with an NK cell line (haNK) expressing the high affinity CD16 allele

**Figure 4:**
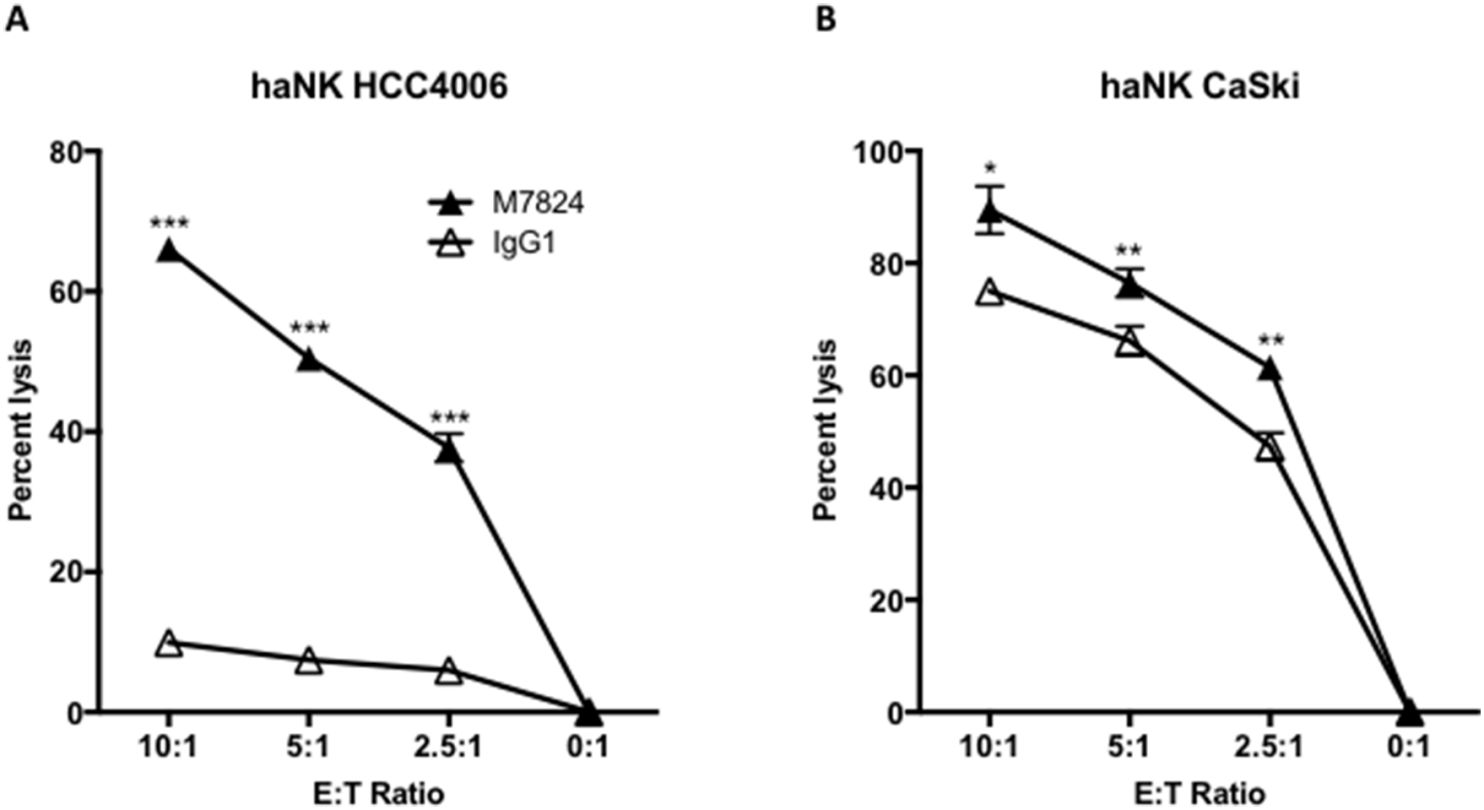
M7824 mediates ADCC of tumor cells employing an NK cell line (haNK) expressing the high affinity CD16 allele Tumor cell lysis mediated by haNK cells as effectors and M7824 (ADCC) were evaluated in 20-hour ^111^In-release assays. haNK cells (irradiated 10 Gy 24 hours prior to the assays) were used as effector cells at different E:T ratios as indicated. Results are shown for Panel **A**: HCC4006 human lung carcinoma cell line, and Panel **B**: CaSki cervical carcinoma cell line, with IgG1 control antibody (1 μg/ml, white triangles) showing haNK lysis, and M7824 (1 μg/ml, black triangles) showing ADCC. Results shown are the averages (SD) of triplicate measurements from one of at least three comparable repeat experiments. Multiple t-tests were used to compare ADCC with haNK cell lysis at each E:T ratio. *** P < 0.001, ** P < 0.01, * *P* < 0.05. Similar results were also observed for the H441 and H460 lung cancer, MDA-MB-231 breast cancer, and PC3 prostate cancer cell lines.

We recently reported [23, 24] that haNK cells, a stable NK cell line that expresses the high affinity CD16 receptor, have the ability to lyse human tumor cells endogenously and by ADCC mediated by anti-PD-L1 MAb and other IgG1 isotype anti-tumor antibodies [23, 24]. We therefore wanted to investigate whether M7824 would also mediate ADCC with haNK cells as effectors. As seen in Figure 4A and 4B, the addition of M7824 increased haNK lysis of two different human carcinoma cell lines at multiple E:T ratios. This enhancement of lysis was also seen with four additional tumor cell lines (H441, H460, MDA-MB-231, and PC3, data not shown).

### Effects of TGFß1 and M7824 on NK cell phenotype and lytic function

**Figure 5:**
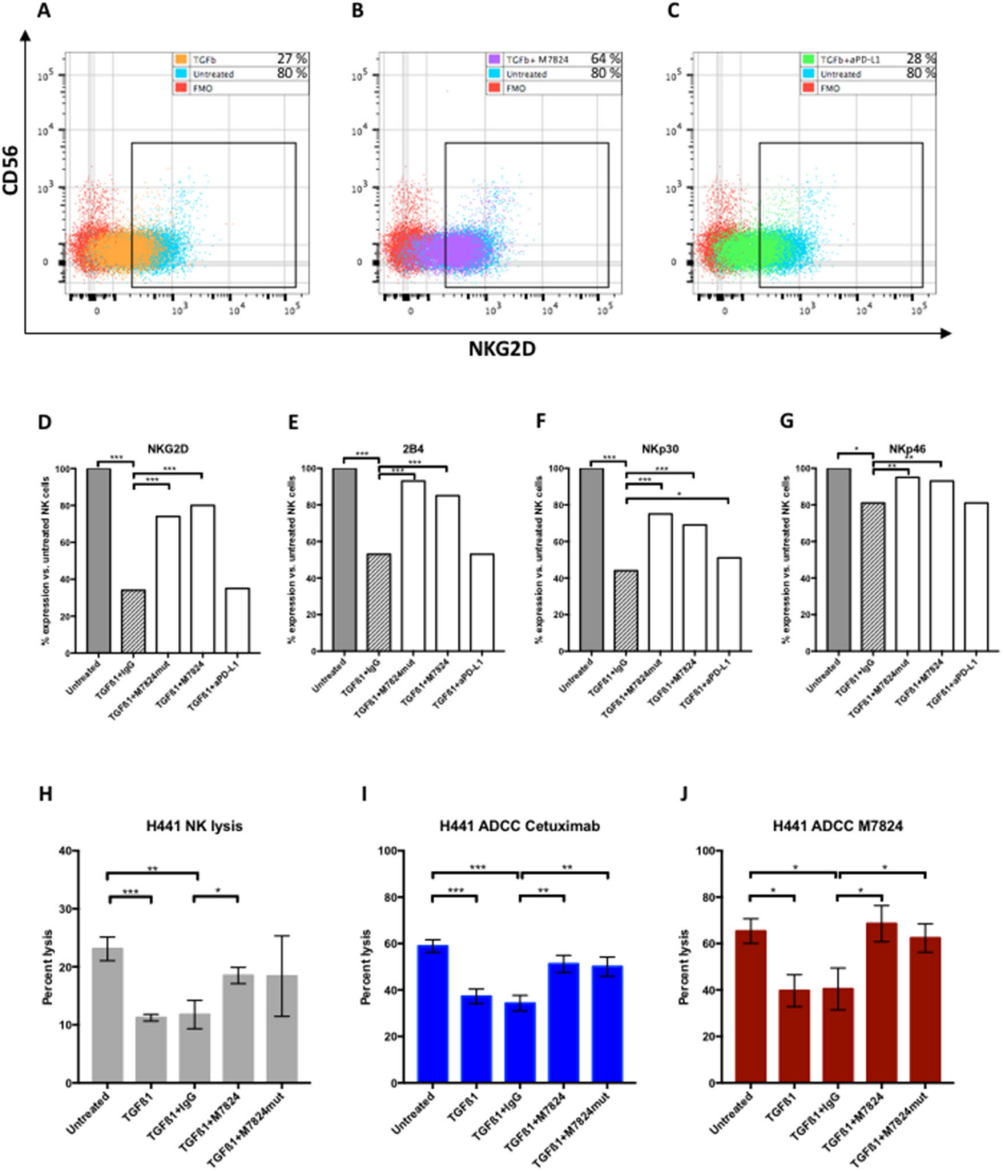
Effects of TGFß1, with and without M7824, on NK cell phenotype and NK lytic function Panels **A-C** NK cells isolated from a healthy donor were incubated for 48 hours with either no treatment, TGFß1, or simultaneously treated with TGFß1 (2 ng/ml) plus isotype control IgG1 MAb, M7824, M7824mut, or anti-PD-L1 MAb; NK cells were then phenotyped by flow cytometry. The results for NKG2D expression for three of the treatment groups are shown using NK cells from one healthy donor in dot plots. Panel A: Untreated and TGFß1-treated NK cells, Panel B: Untreated and TGFß1 plus M7824–treated NK cells, and Panel C: Untreated and TGFß1 plus anti-PD-L1 MAb–treated NK cells. Panels **D-G** The flow cytometry results from NK phenotyping are shown as the change after treatment, by setting the expression level of the markers at 100% for untreated NK cells. Panels **H-J** NK cells isolated from a healthy donor were incubated for 48 hours with no treatment, TGFß1 (2 ng/ml), TGFß1 plus control IgG, TGFß1 plus M7824, or TGFß1 plus M7824mut, and then evaluated in tumor cell lysis assays. Panel H: NK tumor cell lysis (1 μg/ml control IgG1 MAb present in assay); Panel I: ADCC of tumor cells mediated by cetuximab (1 μg/ml); Panel J: ADCC lysis of tumor cells mediated by M7824 (1 μg/ml). In studies shown in Panels H, I and J, after exposure of NK cells to TGFß1 and other treatments, cells were washed prior to use in the ADCC assays. Note different y-axes for Panel H vs. Panels I and J. T-tests were performed to compare untreated NK cells to TGFß1, and TGFß1 to all treatment groups. *** P < 0.001, ** P < 0.01, * *P* < 0.05. Only the significant comparisons are shown.

Prior studies have shown TGFß to be an immunosuppressive entity [25]. Since one component of the M7824 molecule is the TGFßR2, studies were undertaken to determine if the use of M7824, compared to anti-PD-L1, would mitigate or reduce the effect of TGFß on NK-mediated lysis of tumor cells. NK cells were incubated for 48 hours with TGFß1, and then evaluated for changes in phenotype by flow cytometry. Of the 24 markers analyzed (Supplementary Table 2), significant reductions were seen in the expression of NK activation markers NKG2D, 2B4, NKp30, NKp46 and CD122 with the addition of TGFß1 (Figure 5A-5G). As an example, Figure 5A shows the flow cytometry dot plot of NKG2D expression of untreated and TGFß1-treated NK cells. Figure 5B shows the results when M7824 and TGFß1 were simultaneously added to NK cells; Figure 5C shows the results when anti-PD-L1 and TGFß1 were simultaneously added to NK cells. Simultaneous treatment of TGFß1-treated NK cells with either M7824 or the M7824mut (which does not bind to PD-L1) maintained the expression of the NK cell activation markers (Figure 5D-5G); in contrast, when NK cells were simultaneously treated with TGFß1 and anti-PD-L1, the immunosuppressive effects of TGFß1 on NK activation markers was not reversed (Figure 5D-5G). The effect of TGFß1 on NK-mediated tumor cell lysis was also evaluated. As seen in Figure 5H, the reduced lytic capacity of NK cells by TGFß1 could be mitigated in part by the addition of M7824. Cetuximab (Erbitux®) is an anti-epidermal growth factor receptor (EGFR) MAb approved for the treatment of metastatic colorectal cancer and squamous cell carcinoma of the head and neck. Cetuximab is of the IgG1 isotype, and ADCC has been shown to contribute to its clinical efficacy [26, 27]. Therefore, we wanted to examine if treatment of NK cells with TGFß1 would reduce cetuximab-mediated ADCC, and whether M7824 could restore this. As seen in Figure 5I, TGFß1 also reduced the tumor cell lysis by NK cells employing cetuximab to mediate ADCC. Incubating the NK cells with TGFß1 and M7824, then washing, restored most of the ADCC-mediated lytic capacity of cetuximab (Figure 5I) or that mediated by the addition of M7824 (Figure 5J). Moreover, when the M7824mut molecule was incubated with NK cells and TGFß1, the same effects were seen (Figure 5I and 5J). Since the M7824mut molecule has an inactive anti-PD-L1 binding component, these studies indicate that the restoration of NK lytic capacity was due to the TGFßR2 component of the M7824 molecule. Similar results shown in Figure 5 were observed using NK cells from a second donor. In addition, the MDA-MB-231 (breast cancer) and CaSki (cervical cancer) cell lines were evaluated as targets with similar results. Additional experiments were conducted using haNK cells as effectors in the presence or absence of TGFß1. After incubation of haNK cells with TGFß1, cells were washed, and incubated with H441 tumor cells in the presence of either cetuximab or M7824. In both cases, TGFß1 had little or no effect on the endogenous haNK lysis of tumor cells or the ADCC mediated by either MAb (Supplementary Figure 2).

### Effects of TGFß1 and M7824 or anti-PD-L1 on NK cell gene expression

**Figure 6:**
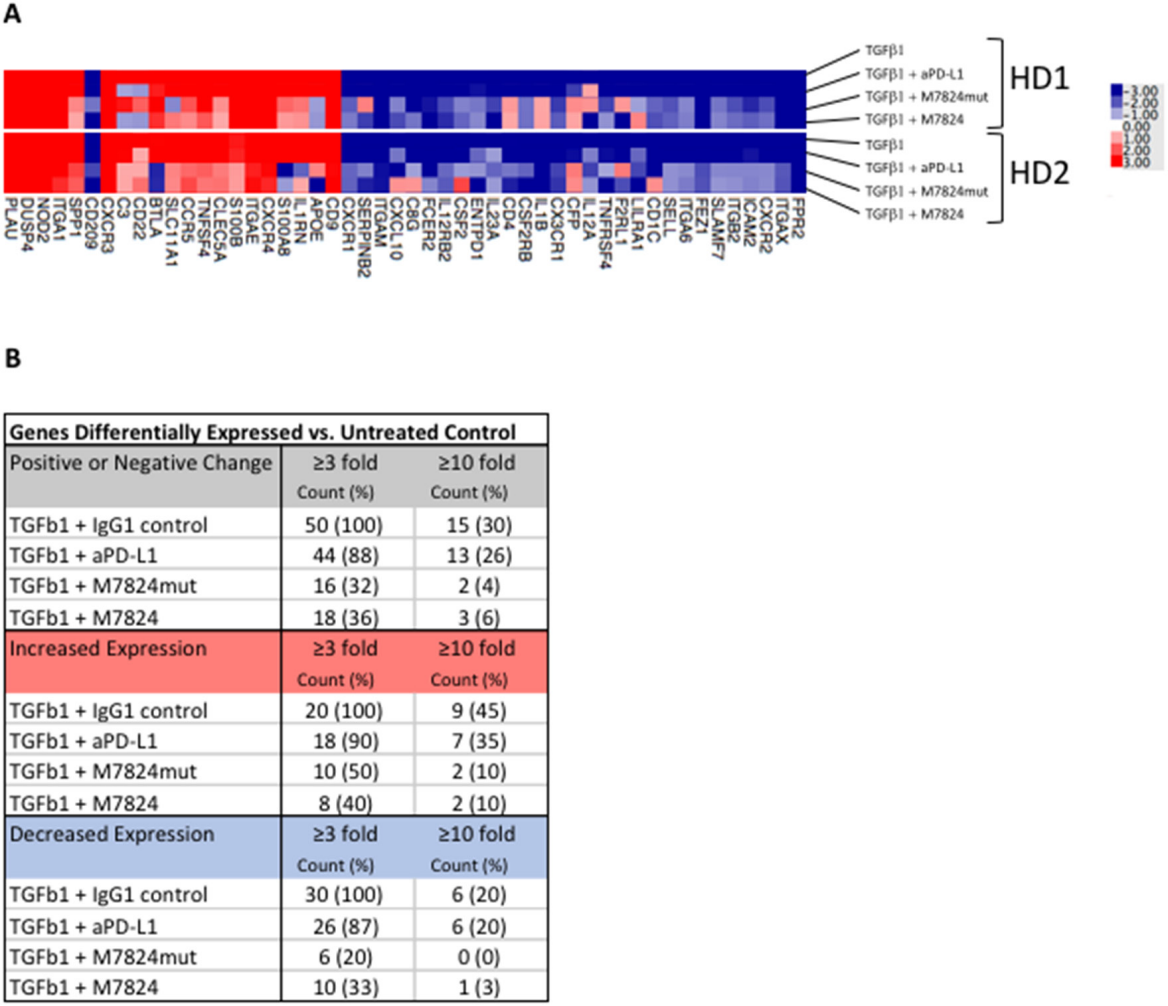
Effects of TGFß1 and M7824 on NK cell gene expression NK cells isolated from 2 healthy donors were incubated for 48 hours with either no treatment, or simultaneously treated with TGFß1 (2 ng/ml) plus isotype control IgG1 MAb (1 μg/ml), M7824 (1 μg/ml), M7824mut (1 μg/ml), or anti-PD-L1 MAb (0.8 μg/ml) prior to RNA extraction for NanoString analysis using the nCounter PanCancer Immune Profiling Panel of 770 immune related genes. Panel **A** A heatmap of the gene expression analysis is shown for the 50/770 genes (6.5%) that were up- or downregulated ≥ 3-fold in both donors after TGFß1-treatment compared to no treatment. Panel **B** Table showing the number of genes differentially expressed vs. untreated control.

We have also conducted NanoString studies to further define the differences between anti-PD-L1 and M7824. As can be seen in Figure 6, of 770 immune related genes evaluated by NanoString technology, 50 genes (6.5%) were up- or downregulated ≥ 3-fold after TGFß1-treatment of NK cells isolated from two healthy donors. Furthermore, 44/50 (88%) of these genes were unaltered when the NK cells were treated simultaneously with TGFß1 and anti-PD-L1, indicating that anti-PD-L1 treatment resulted in minimal modification of gene expression induced by TGFß1. In contrast, in NK cells from both donors that were simultaneously treated with TGFß1 and M7824, the M7824 treatment could partially or completely prevent 32/50 (64%) of the TGFß1-induced gene expression changes; similar results were obtained using the M7824mut, thus providing further support for the anti-TGFß properties of this molecule. The top 10 up- or downregulated genes are shown in Supplementary Table 3.

### Effects of M7824 on Treg suppression of T-cell proliferation

**Figure 7:**
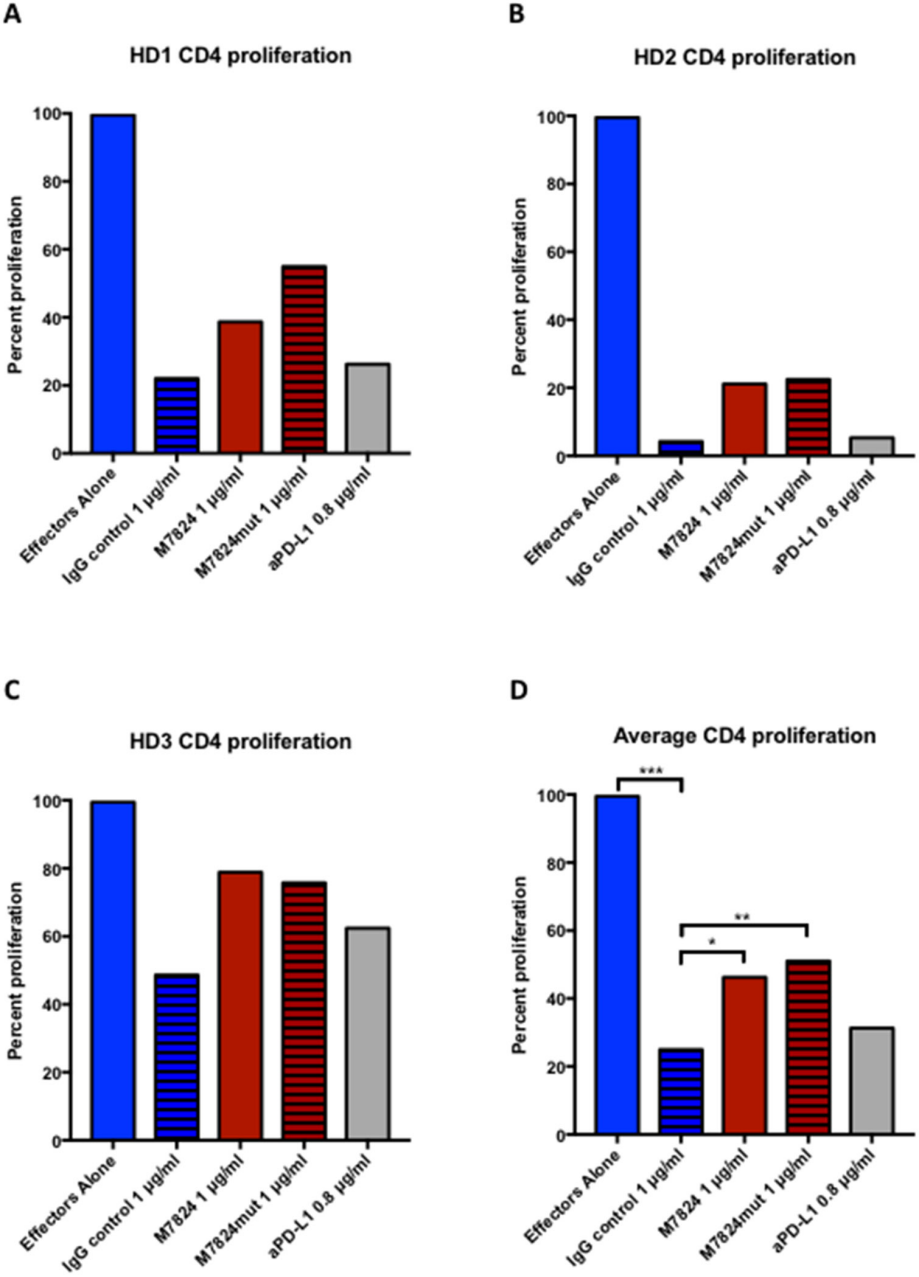
Effects of M7824 on Treg suppression of CD4+ proliferation CD4^+^CD25^NEG/LOW^ effector cells and autologous Tregs (CD4^+^CD25^HIGH^CD127^DIM/NEG^) were isolated from three healthy donors’ PBMCs, and used in a proliferation assay with plate-bound anti-CD3, as described in Materials and Methods. Proliferation of CD4^+^ effectors in the absence of Tregs was set as 100% proliferation. Panels **A-C** show the individual results from the three donors, and Panel **D** shows the average proliferation. In all panels, CD4^+^ effectors alone are shown as blue bars; all other bars indicate CD4^+^ effectors co-incubated with autologous Tregs at a 1:1 ratio with different treatments. The addition of Tregs significantly decreased the CD4^+^ T-cell proliferation (hatched blue bars), and this could be partially restored by treatment with M7824 (red bars) or M7824mut (hatched red bars), but not anti-PD-L1 (gray bars). A t-test was performed to compare the treatment groups to isotype IgG control for the average of all three donors. *** P < 0.001, ** P < 0.01, * *P* < 0.05.

Human Tregs express TGFß, and can thus potentially suppress effector T-cell functions. We therefore wanted to evaluate if exposure of Tregs to M7824 would abrogate this effect. CD4 T cells and Tregs were isolated from three donors. As can be seen in Figure 7, co-incubation of anti-CD3–stimulated CD4 effector cells with autologous Tregs led to decreased proliferation in all three donors. When M7824 or M7824mut was added, the CD4 effector proliferation was partially restored. Since M7824mut also restored CD4 T-cell proliferation, but anti-PD-L1 did not, this effect was due to TGFß1 blockade, and not binding to PD-L1. As seen in Figure 7A-7C, the suppressive ability of the Tregs varied between healthy donors, but for all donors the same trends were seen. Figure 7D shows the results of the average of the three donors.

## DISCUSSION

The studies reported here were aimed to demonstrate the dual functionality of the M7824 agent and to distinguish it from anti-PD-L1 avelumab. While M7824 can clearly mediate ADCC of a range of human tumor cells, on an equimolar basis it is not as potent as anti-PD-L1. ALT-803, an IL-15 superagonist fusion protein complex with better pharmacokinetic and pharmacodynamic properties than recombinant IL-15 [28], increased the ADCC of both agents by enhancing NK activity. The addition of ALT-803, however, also rendered M7824 just as potent as anti-PD-L1 in mediating ADCC of tumor cells at multiple E:T ratios. M7824 mediated ADCC of 7/8 tested human carcinoma cell lines. M7824 did not mediate ADCC of the breast carcinoma cell line MCF7, which could be expected since it does not express PD-L1. These studies also showed no differences in M7824-mediated ADCC using NK effectors from healthy donors or cancer patients, although, as expected, NK activity was shown to vary among different individuals. The NK cells isolated from breast cancer patients used in these studies were obtained before cancer therapy. We have previously compared NK lysis and ADCC mediated by NK effector cells from healthy donors and lung cancer patients [6], and seen no difference in their lytic capacity. It should be noted that these NK cells are from the peripheral blood, and may therefore not display the decreased activity levels of NK cells found in the tumor microenvironment. There may also be differences in NK activity between patients with different cancers, but we have not found that to be the case to date. Studies are planned to determine if M7824 induces ADCC with other types of effectors, such as macrophages; one must consider, however, the differences between subtypes of tumor associated macrophages, and those subtypes of macrophages in the periphery [29, 30].

M7824 was also shown to mediate ADCC of tumor cells using haNK cells as effectors. haNK cells [23, 24, 31, 32] are derived from NK-92 cells, and have been engineered to endogenously express IL-2 and the high affinity CD16 allele. Prior studies have shown [26, 27, 33, 34] that patients receiving the IgG1 MAbs cetuximab, herceptin, and rituximab show enhanced clinical benefit if their NK cells possess the higher affinity CD16 V/V (v, valine) alleles vs. the V/F (F, phenylalanine) or F/F alleles. Unfortunately, only approximately 10-15% of the population possesses the V/V allele [35]. While there are conflicting reports [35, 36] in the literature concerning the contribution of the V/V allele on NK cells to patient benefit, after receiving IgG1 anti-tumor MAbs other confounding factors may be involved, such as the expression of other phenotypic markers on NK cells or the expression of the tumor antigen targets themselves. Larger randomized studies will be required to sort out these results with these IgG1 anti-tumor MAbs, as well as with avelumab and M7824. It should be pointed out that ipilimumab [37] is also an IgG1 MAb.

The functionality of the TGFβR2 portion of M7824 was shown by its ability to mitigate or reduce the phenotypic changes in activation markers on NK cells induced by TGFβ1, as well as its ability to restore the lytic function of NK cells treated with TGFβ1. These activities were shown to be due to the TGFβR2 segment of the molecule, since an identical molecule (M7824mut) with a mutation to abrogate PD-L1 binding also showed these functions, but anti-PD-L1 did not. In contrast to the use of anti-PD-L1, M7824 and M7824mut also had the ability to reduce the suppressive activity of Tregs on CD4^+^ T-cell proliferative activity.

An issue that merits discussion is the rationale for having one bifunctional molecule vs. the use of an anti-PD-L1 in combination with the use of a TGFβ antagonist. TGFβ is a pleiotropic cytokine [15] with normal physiologic functions in different organs, including the control of inflammation and anti-apoptotic activities. The bifunctionality of M7824 is designed to increase the concentration of the TGFβR2 at the site of the tumor and thus the tumor microenvironment via binding to PD-L1 on tumor cells, and also to reduce potential adverse effects of the systemic delivery of a TGFβ antagonist.

A related function of the M7824 agent has recently been detailed in another study [38]. TGFβ1 has been shown to be a driver of the epithelial-to-mesenchymal transition (EMT) process, also termed “mesenchymalization” of carcinoma cells. In that study, treatment of human non-small cell lung cancer cells *in vitro* or as xenografts with M7824 was shown to clearly attenuate features of TGFβ1–mediated mesenchymalization, including a decrease in tumor mesenchymal marker expression and chemotherapy resistance of tumor cells. These changes were not seen with the use of anti-PD-L1.

In conclusion, the multifunctionality of the M7824 molecule in preclinical studies has now been shown as the ability to (a) induce anti-tumor activity in several murine models by inhibiting the binding of PD-1 to PD-L1 (Lan, manuscript submitted), (b) reverse the TGFβ1 induction of mesenchymalization of human carcinoma cells, rendering them more chemo-sensitive [38], and, as shown in this report, (c) mediate ADCC of a range of human carcinoma cells, (d) inhibit the TGFβ1 suppression of NK lysis of tumor cells, and (e) inhibit the suppressive activity of human Tregs on CD4^+^ T cells. A Phase I study of M7824 has recently been completed. Similar adverse events as seen with other anti-PD-1/PD-L1 MAbs were observed, and clear evidence of clinical benefit was seen [17, 18]. Phase II studies employing M7824 in a range of tumor types are ongoing and planned.

## MATERIALS AND METHODS

### Cell lines and cultures

Peripheral blood mononuclear cells (PBMCs) from healthy donors were obtained from the NIH Clinical Center Blood Bank (NCT00001846). PBMCs from breast cancer patients were obtained from a clinical trial at the National Cancer Institute (NCT00179309) [39]. PBMCs were isolated using the LSM Lymphocyte Separation Medium (MP Biomedicals, Santa Ana, CA), washed three times, and frozen at 5x10^7^ cells/ml in 10% DMSO and 90% FCS at -80°C for 24 hours, then moved to liquid nitrogen for storage. Cell counts were performed on a Nexcelom Cellometer Auto 2000 (Nexcelom Bioscience, Lawrence, MA) with AO/PI staining. PBMCs had >95% viability before and after freezing. NK cells from healthy donors and cancer patients were isolated from PBMCs using the Human NK Cell Isolation (negative selection, Kit 130-092-657, Miltenyi Biotech, San Diego, CA) following the manufacturer’s instructions, resulting in > 90% purity. NK cells were rested or treated with TGFß1 (2 ng/ml) or experimental agents in RPMI-1640 supplemented with 10% HsAB (Omega Scientific, Tarzana, CA), 100 U/ml penicillin, 100 μg/ml streptomycin, and 2 mM L-glutamine (Mediatech, Herndon, VA) at 1x10^6^/ml before use in tumor lysis assays. Human tumor cell lines (CaSki: cervical carcinoma; H441: lung carcinoma; H460: lung carcinoma; HCC4006: lung carcinoma; MCF7: breast carcinoma; MDA-MB-231: breast carcinoma; PC3: prostate carcinoma, and SKOV3: ovarian carcinoma) were purchased from American Type Culture Collection (Manassas, VA). All cultures were free of mycoplasma and maintained in RPMI-1640 supplemented with 10% FCS, 100 U/ml penicillin, 100 μg/ml streptomycin, and 2 mM L-glutamine (Mediatech).

### Experimental reagents

The M7824 molecule consists of anti-PD-L1 IgG1 (with structural similarities to avelumab) linked to two TGFßR2. The M7824mut molecule is identical to M7824 with the exception of a mutation in the anti-PD-L1 binding site, which renders it incapable of binding PD-L1 (Lan, manuscript submitted). The M7824, M7824mut, and a matching IgG1 isotype control MAb were obtained from EMD Serono (Rockland, MA) as part of a Cooperative Research and Development Agreement (CRADA) with the National Cancer Institute, NIH. ALT-803 (IL-15 superagonist/IL-15RαSushi-Fc fusion complex [19–21]) was obtained from Altor BioScience (Miramar, FL) as part of a CRADA with the National Cancer Institute, NIH. haNK cells [23, 24] were provided by NantBioScience (Culver City, CA) through a CRADA with the National Cancer Institute, NIH. haNK cells were cultured in phenol free X-Vivo-10 medium (Lonza, Walkersville, MD) supplemented with 5% human heat-inactivated AB serum (Omega Scientific) at a concentration of 5x10^5^/ml. Cetuximab (Bristol-Myers Squibb, Princeton, NJ) was obtained from the NIH Pharmacy.

### NK lysis and ADCC assays

NK cells or irradiated (10 Gy) haNK cells were used as effectors in 20-hour ^111^In-release lysis assays. Target cells were labeled with 20 μCi of ^111^In-oxyquinoline (GE Healthcare, Chicago, IL) at 37°C for 20 minutes and used as targets at 2,000 cells/well in a 96-well round-bottom culture plate at various E:T ratios as indicated. For ADCC experiments, the targets were first incubated for 30 minutes with the test MAb and isotype control MAb before the NK cells were added, as previously described [6]. The plates were incubated for 20 hours at 37°C in a humidified atmosphere containing 5% CO_2_, then harvested and counted on a Wizard^2^ gamma counter (PerkinElmer, Shelton, CT). All samples were tested in triplicate, and specific lysis was calculated from the average. Spontaneous release was determined by incubating targets with medium alone; complete lysis was determined by incubating targets with 0.05% Triton X-100 (Sigma-Aldrich, St. Louis, MO). Specific lysis was determined using the following equation: Percent lysis = (experimental - spontaneous)/(complete - spontaneous) x 100. For the blocking experiments, NK cells were pre-incubated for 2 hours with anti-CD16 antibody (50 μg/ml, clone B73.1, eBioscience, San Diego, CA) before being used in lysis assays with tumor cells at a 10:1 E:T ratio.

### RNA analysis

NK cells isolated from two healthy donors were incubated for 48 hours with either no treatment, or simultaneously treated with TGFß1 (2 ng/ml) plus isotype control IgG1 MAb (1 μg/ml), M7824 (1 μg/ml), M7824mut (1 μg/ml), or anti-PD-L1 MAb (0.8 μg/ml) prior to RNA extraction. Total RNA was extracted from 1x10^6^ cells per sample, using an RNeasy Mini kit, from Qiagen. NanoString analysis of the isolated RNA was completed with the nCounter PanCancer Immune Profiling Panel (NanoString Technologies, Inc., Seattle, WA), run by the Genomics Laboratory, Frederick National Laboratory for Cancer Research, Frederick, MD, as previously described [24]. Raw data files (RCC) and reporter library files (RLF) were uploaded into nSolver analysis software, version 3.0.22 (NanoString). The nCounter Advanced Analysis-Quick Analysis (nSolver, NanoString) was used to analyze the raw data. Normalization of raw data was calculated through the geNorm algorithm, which chose the most consistently expressed housekeeping genes for dataset normalization. Genes that for both donors were upregulated or downregulated more than 3-fold in NK cells treated with TGFß1 compared to control were included in further analyses.

### Regulatory T-cell suppression assay

For Treg suppression assays, PBMC effectors (CD4^+^CD25^NEG^ T cells) and Tregs (CD4^+^CD25^HIGH^CD127^DIM/NEG^) were isolated from healthy donors using the Miltenyi Treg isolation kit 130-094-775 as previously described [40]. Effectors (1 x 10^4^ cells/well) were cultured alone, or co-cultured with autologous Tregs (1 x 10^4^ cells/well) with 0.5 μg/ml of anti-CD3 plate-bound antibody (clone OKT3; eBioscience, San Diego, CA) and irradiated (5,000 rad) T-cell–depleted PBMCs (1 x 10^5^ cell/well) in a 96-well round-bottom plate at 37°C and 5% CO_2_. Cells were cultured in RPMI 1640 complete medium with 10% heat-inactivated human AB serum at a total volume of 200 μl/well. T-cell proliferation was measured by ^3^H-thymidine (PerkinElmer, Waltham, MA) incorporation pulsed on day 4, at 1 μCi (0.037 MBq)/well and quantified 20 hours later using a liquid scintillation counter (PerkinElmer). All experiments were done in triplicate. Proliferation of CD4^+^CD25^NEG^ T cells without co-culturing with CD4^+^CD25^HIGH^CD127^DIM/NEG^ Tregs was defined as 100% proliferation, and the % suppression was calculated.

### Flow cytometric analysis

Flow cytometry of NK cells was performed on a BD LSRII flow cytometer (BD Biosciences, San Jose, CA) and analyzed in FlowJo 9.9 (TreeStar Inc., Ashland, OR). Staining of NK cells was performed with four panels, and the antibodies used were: anti-CD56-BV605, anti-NKG2D-APC, anti-CD107a-PE-Cy7, anti-GranzymeB-FITC, anti-perforin-APC, anti-NKp46-PE-Cy7, anti-NKp30-APC, anti-NKp44-PE, anti-2B4-FITC, anti-4-1BB-PerCP-Cy5.5, anti-CD95-BV421, anti-Tim3-BV421, anti-TRAIL-PE, anti-CD122-PerCP-Cy5.5, anti-CD122-BV510, anti-CD95L-PE, anti-Ki67-BV421, anti-CD40L-BV510, anti-PD-1-PE-Cy7; all were obtained from BioLegend (San Diego, CA). Anti-CD16-APC-H7, PD-L1-PE-Cy7, and CD11a-FITC, were obtained from BD Biosciences (San Jose, CA). Anti-NKG2A-PE was from R&D Systems (Minneapolis, MN), and anti-CD158a-PerCP-Cy5.5 from eBioscience.

### Statistical analyses

Statistical analyses were performed in GraphPad Prism 7 (GraphPad Software, La Jolla, CA), using multiple t-tests, with a desired false discovery rate of 1.00%.

## SUPPLEMENTARY MATERIALS TABLES AND FIGURES



## Abbreviations

ADCC: antibody-dependent cell-mediated cytotoxicity
CRADA: Cooperative Research and Development Agreement
E:T: Teffector to target cell
EMT: epithelial-to-mesenchymal transition
F: phenylalanine
MAb: monoclonal antibody
NK: natural killer
NCI: National Cancer Institute
NIH: National Institutes of Health
PBMC: peripheral blood mononuclear cell
TGFβR2: TGFβ receptor 2
Tregs: regulatory T cells
V: valine
