# Supplementary material for: Analyses of functions of an anti-PD-L1/TGFβR2 bispecific fusion protein (M7824)
Source: Oncotarget. 2017 Sep 8;8(43):75217–31. doi: 10.18632/oncotarget.20680 (PMC5650414; doi:10.18632/oncotarget.20680)
Supplement: Supplementary file 1 [file oncotarget-08-75217-s001.pdf]

Analyses of functions of an anti-PD-L1/TGFβR2 bispecific fusion protein (M7824)

SUPPLEMENTARY MATERIALS

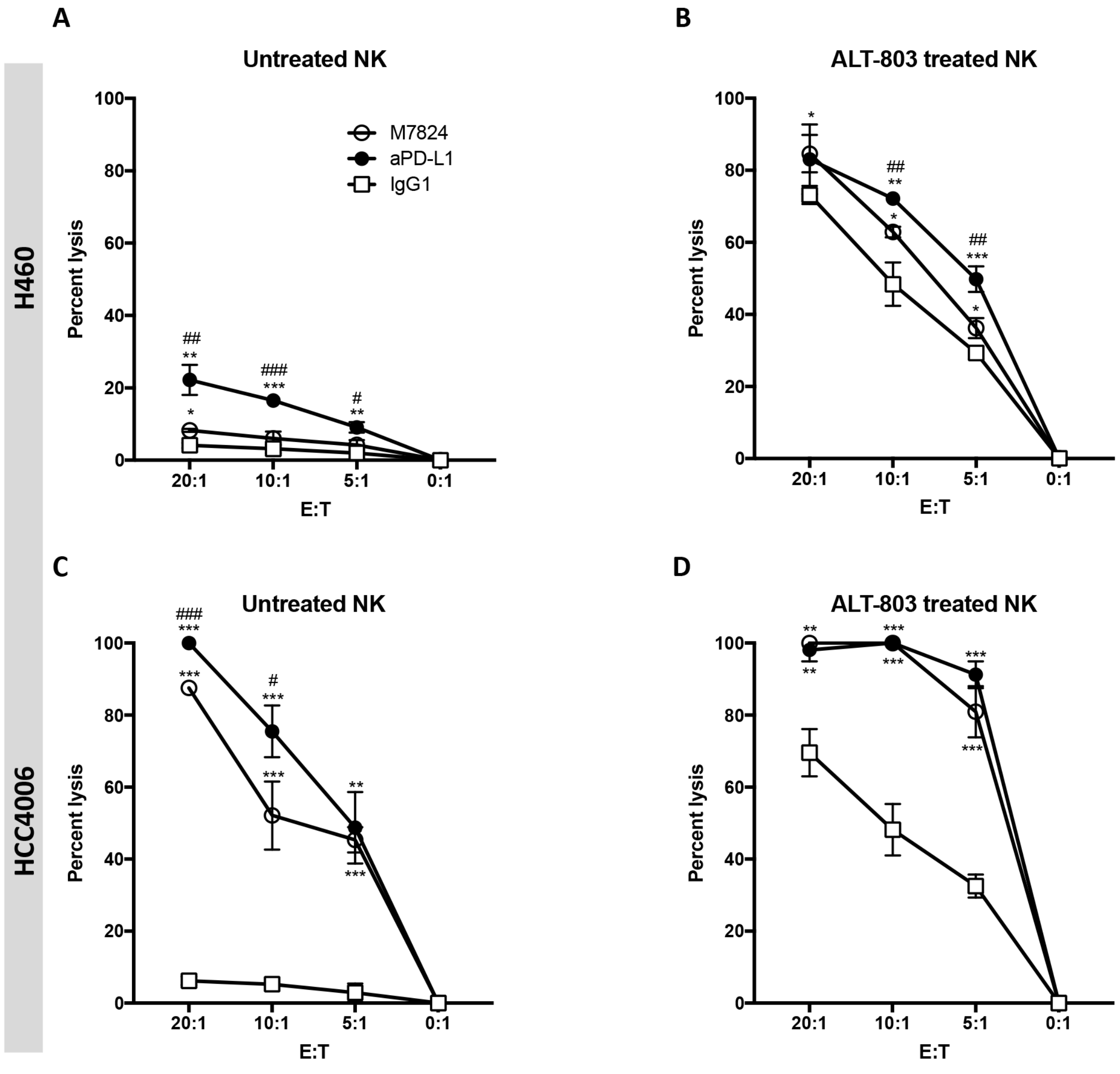

Supplementary Figure 1: Comparison of ADCC of tumor cells mediated by M7824 or anti-PD-L1 with and without pre-treatment of the NK cells with ALT-803. NK cells were isolated from PBMCs from 3 healthy donors, rested untreated or treated (24 h) with ALT-803 (IL-15 superagonist/IL-15RαSushi-Fc fusion complex, 25 ng/ml), and then used in ^{111}In-release 20h assays to evaluate NK tumor cell lysis (white squares, IgG1 isotype control, 1 µg/ml) or ADCC of tumor cells mediated by M7824 (white circles, 1 µg/ml) or anti-PD-L1 (black circles, 0.8 µg/ml) as described in Materials and Methods. Only control IgG1 antibody is shown, since the no MAb control overlapped. Results from 1 out of 3 healthy donors are shown, with mean and standard deviations of triplicate wells for lung carcinoma cell lines H460 (panels **A** and **B**), and HCC4006 (panels **C** and **D**). NK cells from the other 2 healthy donors showed similar results. Multiple t-tests were used to compare ADCC mediated by anti-PD-L1 (black circles) vs. NK lysis (white squares), and ADCC mediated by M7824 (white circles) vs. NK lysis (white squares), at each E:T ratio, *** $P < 0.001$, ** $P < 0.01$, * $P < 0.05$. In addition, we also compared ADCC mediated by M7824 (white circles) vs. anti-PD-L1 (black circles), $^{###}$ $P < 0.001$, $^{##}$ $P < 0.01$, $^{\#}P < 0.05$. Only the significant comparisons are shown.

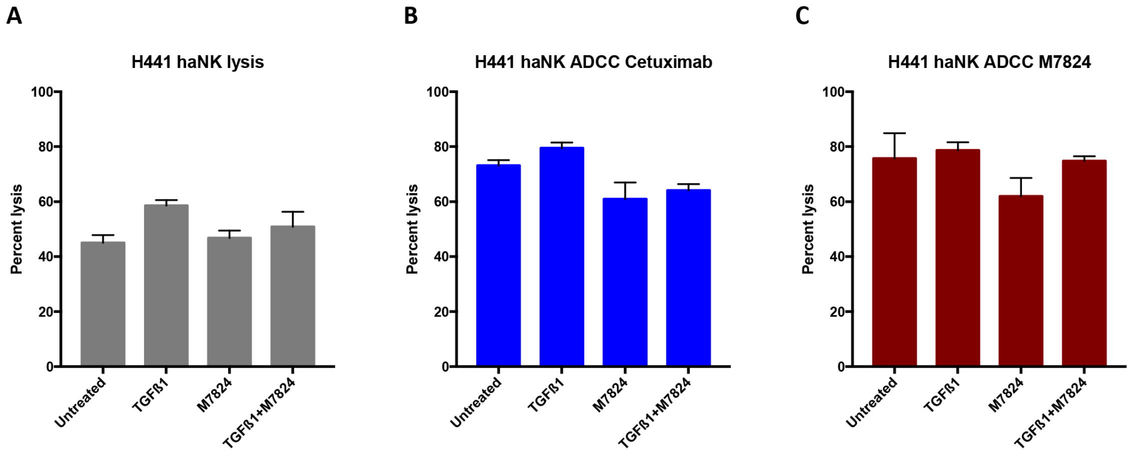

Supplementary Figure 2: Effects of TGFß1 and M7824 on haNK cell tumor lysis. haNK cells were incubated for 48 h with no treatment, TGFß1 (2 ng/ml), M7824 (1 µg/ml), or TGFß1 plus M7824, and then used in a 20-h ^{111}In-release tumor lysis assay to evaluate the effects of treatment on **A** haNK lysis (1 µg/ml IgG1 Mab present in assay), **B** ADCC mediated by cetuximab (1 µg/ml), and **C** ADCC mediated by M7824 (1 µg/ml). The results are shown for tumor lysis of H441 (human lung cancer cell line) at an E:T ratio of 10:1, and have been repeated multiple times.

Supplementary Table 1: NK tumor cell lysis and ADCC tumor cell lysis mediated by M7824 using NK cells isolated from 8 healthy donors

Cell line	Cancer type	% NK lysis (median and IQR)	% ADCC induced by M7824 (median and IQR)	P-value (Mann-Whitney)
H441	Lung	7.2 (4.6-13.8)	52.2 (40.7-71.4)	0.0006
HCC4006	Lung	2.9 (1.6-3.9)	33.4 (17.4-43.5)	0.0104
H460	Lung	5.5 (4.0-6.6)	10.3 (6.5-15.9)	0.0002
MDA-MB-231	Breast	2.5 (1.8-3.9)	29.7 (20.3-33.0)	0.0002
MCF7	Breast	30.3 (18.0-48.6)	32.3 (21.8-46.8)	n.s.
PC3	Prostate	4.3 (1.2-5.8)	22.1 (15.9-27.7)	0.0011
CaSki	Cervical	10.8 (5.4-14.3)	48.2 (43.8-60.7)	0.0003

NK cells isolated from the PBMCs of 8 healthy donors were rested overnight, and then used in an ^{111}In-release 20-h assay to evaluate NK tumor cell lysis (no MAb and IgG1 1 µg/ml) and ADCC tumor cell lysis mediated by M7824 (1 µg/ml), as described in Materials and Methods. Results are shown for the median (IQR) % target lysis at an E:T ratio of 20:1 for all 8 healthy donors assayed in triplicate wells. A Mann-Whitney t-test was performed to compare lysis in wells with IgG vs. wells with M7824. E:T ratios of 10:1 and 5:1 were also evaluated with similar results.
n.s., not significant.

Supplementary Table 2: Phenotypic changes of NK cells treated with TGFß1 with and without M7824, M7824mut or anti-PD-L1

	NKG2D+		2B4+		NKp30+		NKp46+		CD122+		GrZ B+		Perforin+	
	**HD1**	**HD2**	**HD1**	**HD2**	**HD1**	**HD2**	**HD1**	**HD2**	**HD1**	**HD2**	**HD1**	**HD2**	**HD1**	**HD2**
Untreated	80	82	62.1	69.7	16	59	76	88	90	94	95	92	78	77
TGFß1 + IgG1	27	35	23.5	37	2.4	26	61	71	75	82	94	91	70	72
TGFß1 + M7824mut	59	71	43.1	64.8	4.3	44	73	84	89	93	94	92	73	78
TGFß1 + M7824	64	65	46	59	5.2	41	74	82	90	91	94	92	76	70
TGFß1 + anti-PD-L1	28	35	25.9	36.8	2.5	30	59	71	75	81	93	92	73	63

NK cells isolated from 2 healthy donors were incubated for 48 h with no treatment, or treated with TGFß1 (2 ng/ml) with or without IgG1 isotype control MAb (1 µg/ml), M7824mut (1 µg/ml), M7824 (1 µg/ml), or anti-PD-L1 (0.8 µg/ml), and then evaluated by flow cytometry, as described in Materials and Methods. NK cell markers used were: CD16, 2B4, NKG2A, NKG2D, NKp30, NKp44, NKp46, CD122, CD107a, GrZ B, Perforin, Ki67, CD11a, CD158a, CD158b, 4-1BB, CD27, CD40L, CD95 (FAS), FASL, PD-L1, Tim3, CD25, and TRAIL. Only 2B4, NKG2D, NKp30, NKp46 and CD122 changed significantly.

Supplementary Table 3: The 10 most up- or downregulated genes in NK cells treated with TGFß1 and M7824, M7824mut or anti-PD-L1

Gene ID	Gene Name	Properties	Diseases associated with gene
PLAU	Plasminogen activator, urokinase	Secreted serine protease that converts plasminogen to plasmin	Alzheimer's, Quebec platelet disorder
DUSP4	Dual specificity phosphatase 4	Inactivates ERK1, ERK2 and JNK, is expressed in a variety of tissues, and is localized in the nucleus	Echolalia, chronic laryngitis
SPP1	Secreted phosphoprotein 1	Cytokine that upregulates expression of IFNg and IL-12	Malignant pleural mesothelioma, pediatric systemic lupus erythematosus
ITGA1	Fibrinogen beta chain	Beta component of fibrinogen, cleavage products regulate cell adhesion and spreading, display vasoconstrictor/ chemotactic activities	Afibrinogenemia, dysfibrinogenemia, hypodysfibrinogenemia, thrombotic tendency
NOD2	Nucleotide binding oligomerization domain-containing protein 2	Plays a role in immune response to LPS, activates NFKB protein; encodes 2 capase recruitment domains and 6 leucine-rich repeats	Crohn’s disease, Blau syndrome
APOE	Apolipoprotein E	Binds to a specific liver and peripheral cell receptor	Familial dysbetalipoproteinemia, or type III hyperlipoproteinemia (HLP III)
CSF2	Colony stimulating factor 2	GMCSF; cytokine that controls the production, differentiation, and function of granulocytes and macrophages	5q-syndrome, acute myelogenous leukemia
SERPINB2	Serpin family B member 2	Related to the clotting cascade pathway and the senescence and autophagy in cancer pathway	Lipodermatosclerosis, gingivitis
ITGAM	Integrin subunit alpha M	Part of a leukocyte-specific integrin referred to as macrophage receptor 1; important in the adherence of neutrophils and monocytes to stimulated endothelium, and in phagocytosis of complement-coated particles	Systemic lupus erythematosus
ENTPD1	Ectonucleoside triphosphate diphosphohydrolase 1	Plasma membrane protein that hydrolyzes extracellular ATP and ADP to AMP; inhibition may confer anticancer benefits	Spastic paraplegia 64

Annotations for the 10 most up- or downregulated genes with information from GeneCards Human Gene Database. NK cells isolated from 2 healthy donors were incubated for 48 hours with either no treatment, or simultaneously treated with TGFß1 (2 ng/ml) plus isotype control IgG1 MAb (1 µg/ml), M7824 (1 µg/ml), M7824mut (1 µg/ml), or anti-PD-L1 MAb (0.8 µg/ml) prior to RNA extraction for NanoString analysis of 770 immune related genes.
